# Silica Nanoparticle-Reinforced Wormlike Micellar Gels for High-Temperature Flow Redistribution in Heterogeneous Porous Media

**DOI:** 10.3390/gels12080731

**Published:** 2026-08-17

**Authors:** Kun Zhang, Xiongfei Liu

**Affiliations:** State Key Laboratory of Petroleum Resources and Engineering, China University of Petroleum (Beijing), Beijing 102249, China

**Keywords:** wormlike micelles, viscoelastic network, in situ gelation, nanoparticle size effect, flow modulation, high-temperature reservoir

## Abstract

Maintaining the rheological performance of wormlike micellar fluids at elevated temperatures remains challenging. Conventional viscoelastic surfactant (VES) systems may undergo thermally induced micellar scission and loss of gel-like viscoelasticity at elevated temperatures. In this study, we investigate the size-dependent reinforcement of long-chain C22^+^ wormlike micellar systems by silica nanoparticles under a temperature-ramp protocol reaching 160 °C. Under the applied temperature-ramp protocol, the formulation containing 0.10 wt% of 15 nm SiO_2_ nanoparticles exhibited the highest measured rheological response among the tested formulations, retaining an apparent viscosity of approximately 210 mPa·s and a plateau storage modulus of approximately 18.5 Pa during the 20 min isothermal holding period at 160 °C, compared with a plateau storage modulus of approximately 11 Pa for the corresponding VES system. At equal nanoparticle mass loading, the 15 nm particles produced approximately 18% and 6% higher G′ and G″, respectively, than the 500 nm particles. The rheological results, together with qualitative electrokinetic measurements after dilution, are consistent with nanoparticle-surfactant association that may promote micellar entanglement and network reinforcement. The nanoparticle-enhanced viscoelastic surfactant (N-EVES) formulation reduced the acid-rock reaction rate to approximately 25% of that measured for conventional HCl while showing an apparent effective H^+^ diffusion coefficient of the same order. Scanning electron microscopy–energy-dispersive X-ray spectroscopy (SEM–EDS) detected Si- and N-containing species on the treated carbonate surface, suggesting that surface adsorption or deposition may contribute to reaction retardation. Parallel dual-core flooding under a permeability contrast of approximately 13 showed fluid redistribution toward the low-permeability core. Based on the axial wormhole penetration length obtained from the CT reconstruction, the normalized axial wormhole penetration fraction of the low-permeability core was approximately 70% for the 0.10 wt% formulation. These results provide experimental evidence of nanoparticle-size-dependent rheological reinforcement, acid-rock reaction retardation, and core-scale flow redistribution under strongly acidic and high-temperature conditions.

## 1. Introduction

Supramolecular self-assembly based on viscoelastic surfactants (VES) has garnered tremendous attention in soft matter and gel science due to its fascinating stimulus-responsive properties and broad applications in rheology modification and complex fluid engineering [1,2]. In aqueous solutions, surfactant molecules spontaneously assemble into one-dimensional (1D) wormlike micelles (WLMs), which further entangle to form transient three-dimensional (3D) networks, imparting viscoelasticity to the system [1,2]. Wormlike micelles are commonly described as “living polymers” because reversible micellar scission and recombination govern their characteristic relaxation behavior. Their contour length, entanglement density, and viscoelastic response depend strongly on surfactant concentration, counterions, temperature, and solution composition [3,4,5,6]. At elevated temperatures, increased molecular motion can disrupt hydrophobic association and accelerate micellar scission. In many reported VES systems, pronounced reductions in viscosity and elasticity occur above approximately 140 °C [7,8,9].

This temperature sensitivity is particularly relevant to the acidizing stimulation of deep and ultra-deep carbonate reservoirs [10,11,12]. These reservoirs commonly exhibit high temperatures and pronounced permeability heterogeneity, both of which complicate acid placement [12,13]. A reduction in the viscosity and elasticity of the VES network can decrease flow resistance and diversion capacity, thereby promoting preferential acid flow through high-permeability pathways and limiting acid access to lower-permeability regions [14,15,16]. Consequently, there is a practical need for VES formulations that retain or develop sufficient flow resistance under high-temperature acidizing conditions while maintaining effective acid transport and carbonate dissolution [8,17,18].

The incorporation of inorganic nanoparticles (NPs) has been investigated as a strategy for modifying the rheological behavior of supramolecular micellar systems [19]. NPs may interact with micellar aggregates through electrostatic interactions, surface adsorption, spatial confinement, or physical association, thereby affecting micellar growth, entanglement, and relaxation behavior [20]. Previous work on silica-containing wormlike micellar systems has shown that moderate silica loading can increase zero-shear viscosity and plateau modulus, whereas further increases in particle loading may reduce the rheological response because of changes in surfactant adsorption and aggregate structure [21]. More broadly, nanoparticle-induced rheological modification depends on particle size, concentration, surface chemistry, surfactant structure, and solution conditions [22,23]. However, the particle-size-dependent behavior of nanoparticle-enhanced WLM systems under strongly acidic and high-temperature conditions remains insufficiently characterized. It also remains unclear whether such rheological differences are accompanied by measurable changes in carbonate reaction kinetics, surface adsorption or deposition, and flow redistribution in heterogeneous porous media [24].

Accordingly, this study evaluates a long-chain C22^+^ nanoparticle-enhanced viscoelastic surfactant (N-EVES) acid system, containing silica nanoparticles with different nominal diameters. High-temperature steady-shear and oscillatory rheological measurements were used to determine the effects of nanoparticle size and concentration on the bulk rheological response under a temperature-ramp protocol reaching 160 °C. Diluted-sample zeta-potential measurements were used as qualitative indicators of nanoparticle-surfactant interactions. Rotating-disk experiments and SEM-EDS analysis were employed to examine acid-rock reaction retardation and surface adsorption or deposition. Single-core and parallel dual-core flooding experiments, combined with 3D-CT reconstruction, were then used to evaluate pressure response, fluid redistribution, and post-treatment wormhole morphology in carbonate cores with different permeabilities. By linking particle-size-dependent rheology with interfacial reaction behavior and core-scale fluid allocation, this study provides an integrated experimental assessment of silica-nanoparticle-enhanced WLM fluids under strongly acidic and high-temperature conditions.

## 2. Results and Discussion

### 2.1. Microscopic Morphology of Nanoparticles and N-EVES Colloids

**Figure 1:**
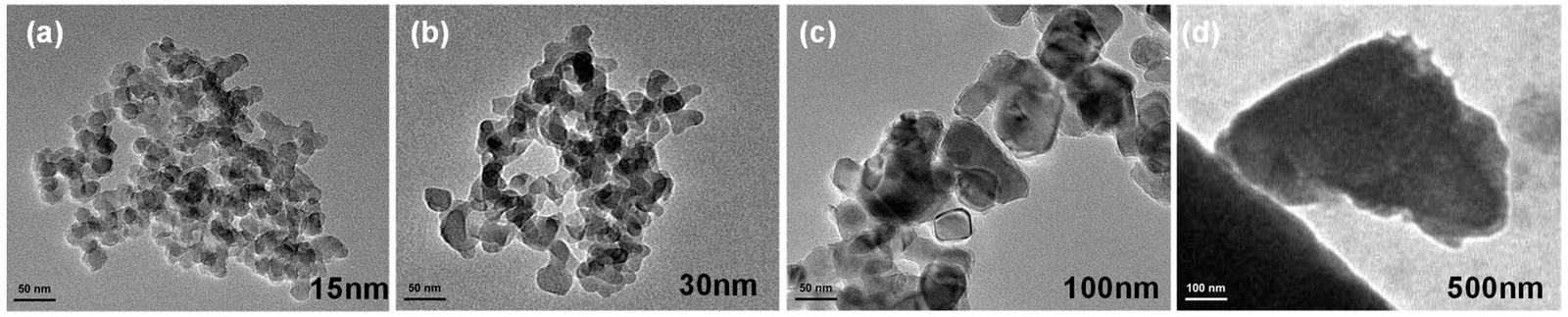
Transmission electron microscopy images showing the size and aggregation morphologies of hydrophilic SiO_2_ nanoparticles: (**a**) 15 nm, (**b**) 30 nm, (**c**) 100 nm, and (**d**) 500 nm.

Before investigating the high-temperature rheological behavior, TEM was used to examine the morphologies of SiO_2_ nanoparticles with different nominal sizes and the dried N-EVES aggregates. As illustrated in Figure 1a–d, the four distinct nanoparticle sizes exhibit different particle sizes and aggregation morphologies. The 15 and 30 nm nanoparticles exhibit relatively small, near-spherical primary particles together with some soft agglomeration, which may be associated with their larger surface-area-to-volume ratios. The 100 nm and 500 nm samples exhibit larger particle dimensions and aggregates. These differences in nominal size and aggregation morphology may affect their interactions with the VES system.

**Figure 2:**
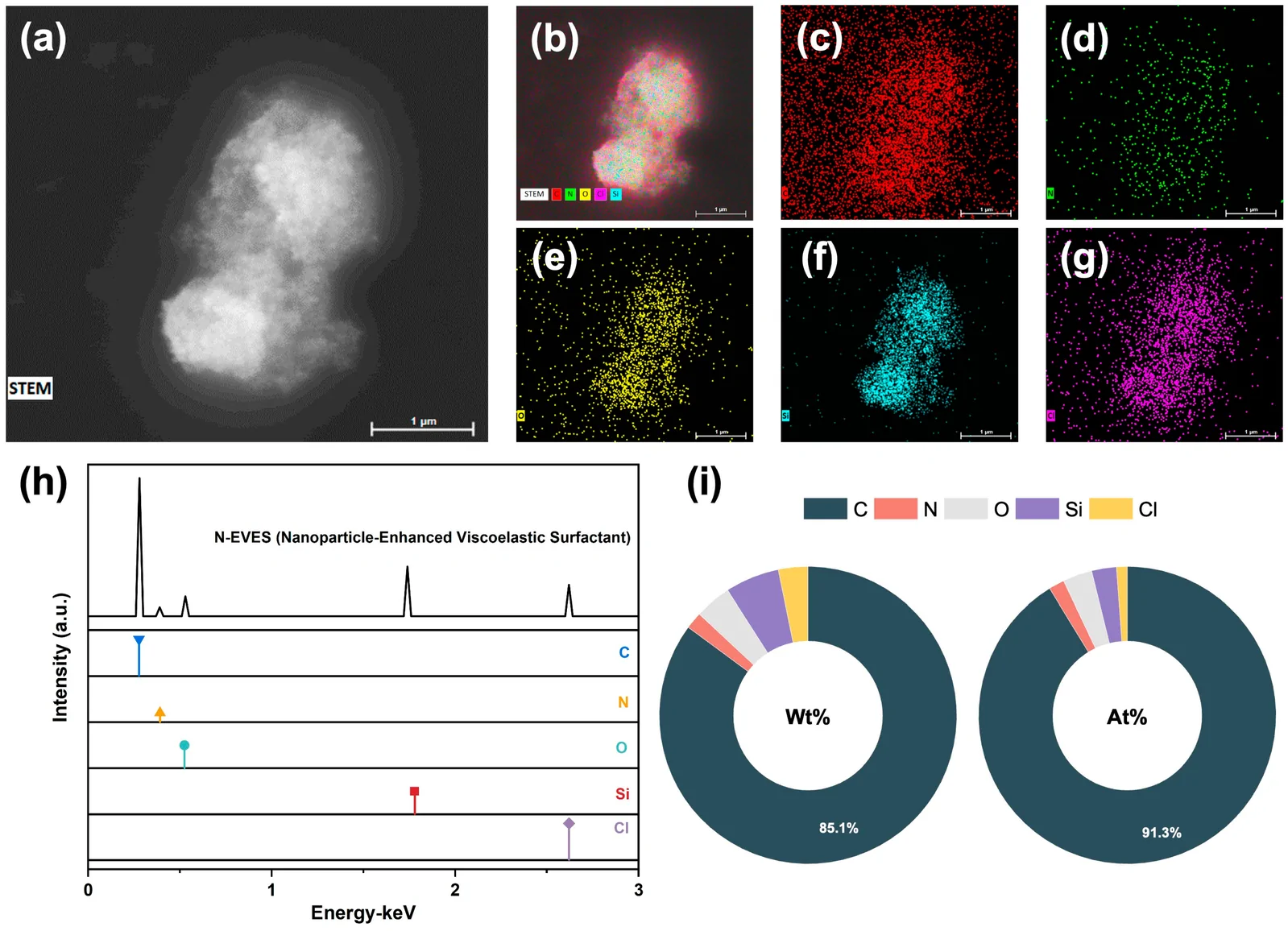
Microscopic morphology and elemental characterization of the dried N-EVES aggregates: (**a**) HAADF-STEM image, (**b**–**g**) corresponding EDS elemental mapping of C, N, O, Si, and Cl, and (**h**,**i**) EDS spectra and quantitative elemental composition, showing the spatial co-localization of silica nanoparticles and VES-derived organic components within the dried colloidal aggregates.

The colloidal morphology and elemental distribution of the N-EVES system were further characterized using STEM and EDS mapping. The STEM images show aggregates containing both the VES-derived organic components and 15 nm SiO_2_ nanoparticles. Elemental mapping shows spatial overlap between the Si- and O-containing regions and the C- and N-containing regions (Figure 2b–g). Furthermore, the semi-quantitative EDS results in Figure 2h,i show that carbon remains the dominant element (with a weight fraction of 85.1%), accompanied by the emergence of characteristic Si signals. The spatial coincidence of the organic and inorganic elemental signals supports association between the VES components and SiO_2_ nanoparticles. However, the dried STEM-EDS observations do not directly demonstrate nanoparticle bridging between individual wormlike micelles.

### 2.2. Rheological and Viscoelastic Properties at High Temperatures

#### 2.2.1. High-Temperature Rheological Response

**Figure 3:**
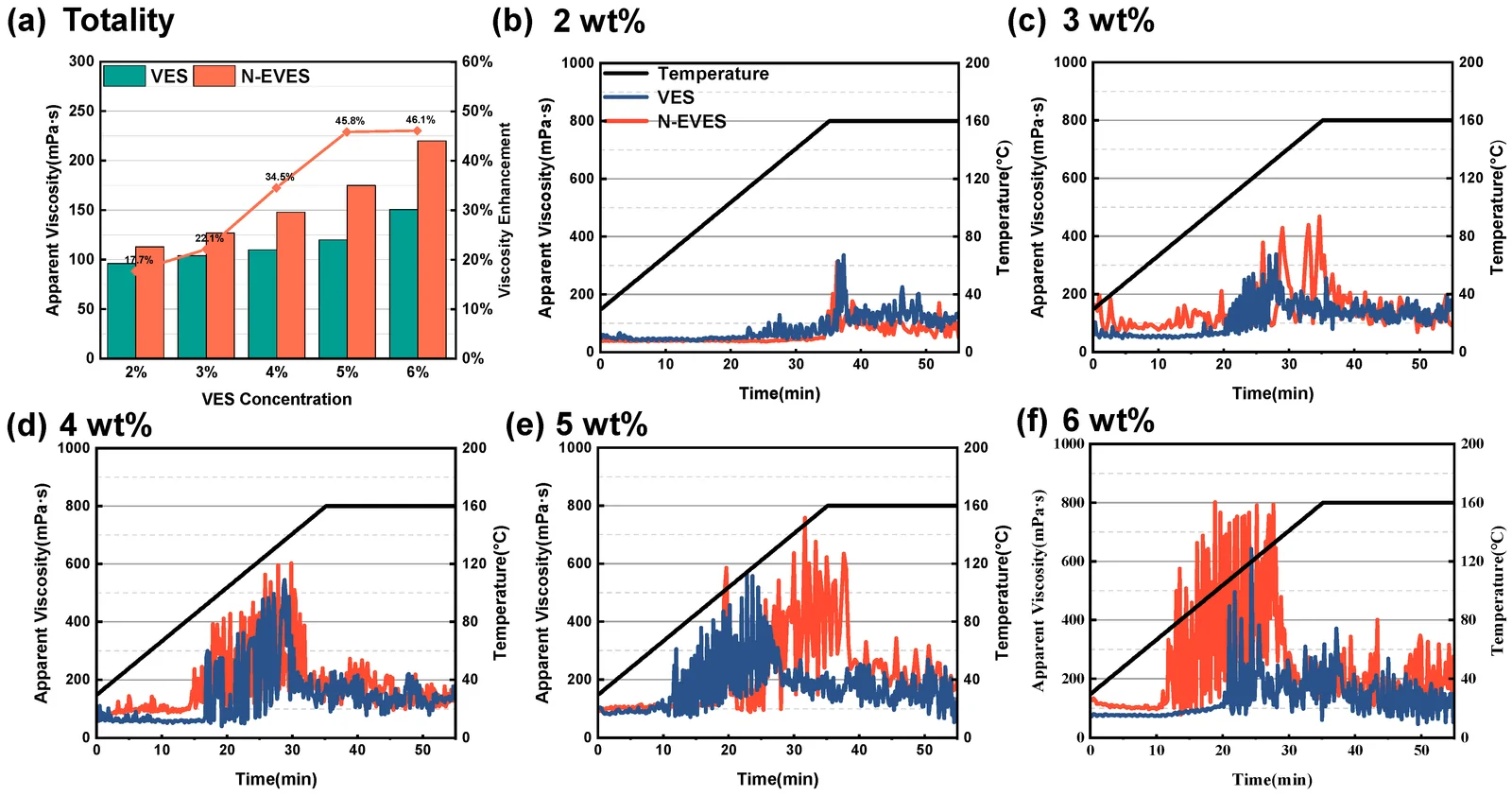
Rheological behavior and high-temperature rheological response of the pure VES and N-EVES systems at varying surfactant concentrations (2 wt–6 wt%): (**a**) mean apparent viscosity during the 20 min isothermal holding period at 160 °C and corresponding viscosity enhancement ratio at 160 °C; (**b**–**f**) dynamic apparent viscosity evolution during the temperature ramp-up process. In all tests, the HCl concentration was 15 wt%, the corrosion-inhibitor concentration was 0.50 wt%, and the 15 nm SiO_2_ concentration in the N-EVES systems was 0.10 wt%; only the formulated VES concentration was varied.

The macroscopic diverting capacity of acid systems in high-temperature reservoirs is strongly influenced by their apparent-viscosity response during heating. Steady-shear viscosity curves obtained during a temperature ramp (from 30 °C to 160 °C) show an initial increase followed by a decrease in apparent viscosity (Figure 3b–f). Initially, as the temperature increases, a prominent viscosity peak (exceeding 500 mPa·s in some cases) is observed, which may reflect temperature-dependent micellar growth and structural reorganization. However, upon reaching 160 °C, the apparent viscosity of the VES control decreases substantially, which may be associated with thermally induced micellar scission. The N-EVES system retained a higher apparent viscosity than the VES control upon reaching 160 °C under the applied temperature-ramp protocol. As depicted in the viscosity enhancement bar charts (Figure 3a), the incorporation of nanoparticles increased the apparent viscosity recorded upon reaching 160 °C. Furthermore, this enhancement effect shows a concentration-dependent trend: as the VES concentration increases from 2 wt% to 6 wt%, the viscosity enhancement ratio monotonically increases from 17.7% to a substantial 46.1%. This trend is consistent with a stronger nanoparticle-associated reinforcement effect at higher VES concentrations; however, the underlying molecular mechanism was not directly determined.

During the temperature ramp, transient viscosity fluctuations were observed, which may reflect transient sample restructuring and measurement-related effects under simultaneous heating and shear.

#### 2.2.2. Effects of Nanoparticle Concentration and Size on Rheological Properties

**Figure 4:**
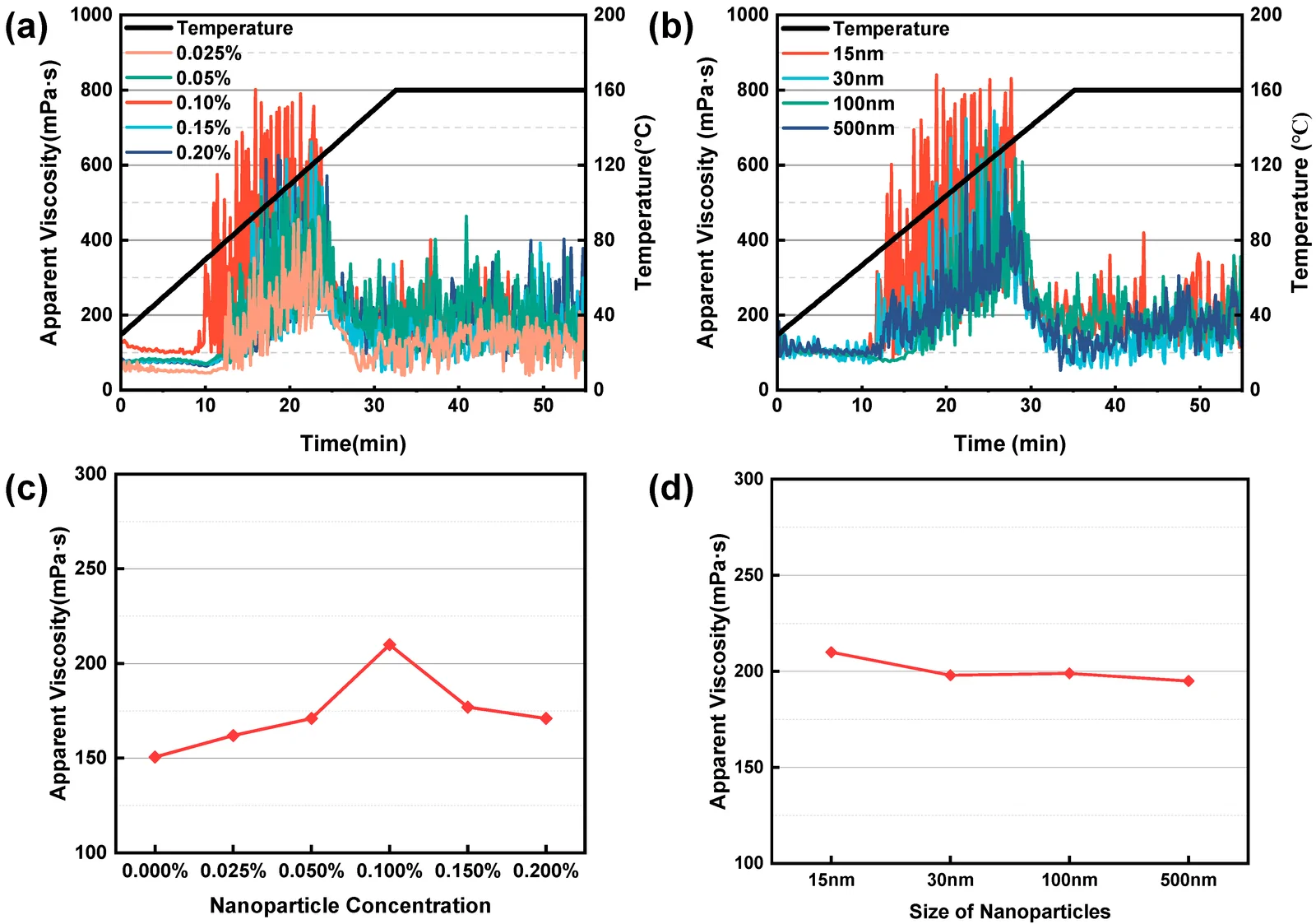
Influence of silica nanoparticles on the rheological properties and high-temperature viscosity response of the VES systems: (**a**) dynamic apparent viscosity as a function of nanoparticle concentration during the temperature ramp-up, (**b**) effect of nanoparticle size on the dynamic viscosity profiles, (**c**) apparent viscosity at 160 °C versus nanoparticle concentration, and (**d**) correlation between steady-state viscosity and nanoparticle size. All formulations contained 15 wt% HCl, 6 wt% formulated VES solution, and 0.50 wt% corrosion inhibitor. In panels (**a**,**c**), the 15 nm SiO_2_ concentration was varied; in panels (**b**,**d**), the SiO_2_ concentration was fixed at 0.10 wt% and only the nominal particle size was varied.

To evaluate the effects of nanoparticle concentration and size on the high-temperature rheological behavior of the N-EVES system, the concentration and size of the SiO_2_ nanoparticles were systematically varied. The apparent viscosity profiles at 160 °C (Figure 4a,c) show that the highest measured value, approximately 210 mPa·s, was obtained at a nanoparticle concentration of 0.10 wt%. Below 0.10 wt%, the measured viscosity was lower, whereas further increasing the concentration to 0.15 and 0.20 wt% also resulted in a decrease. This non-monotonic behavior may be related to changes in nanoparticle-surfactant association, particle aggregation, or micellar organization, although these contributions were not independently resolved.

Furthermore, the nanoparticle size effect influences the rheological behavior. At the dosage of 0.10 wt%, reducing the nanoparticle diameter from 500 nm down to 15 nm produces a monotonic increase in the apparent viscosity at 160 °C (Figure 4b,d). At equal mass loading, the smaller nanoparticles are expected to provide a larger total geometric surface area and more potential interfacial interaction sites. This difference may contribute to the stronger rheological enhancement observed for the 15 nm particles. Because particle size and total available surface area varied simultaneously at equal mass concentration, their individual contributions cannot be completely separated in the present comparison.

The rheological results demonstrate that the formulated N-EVES system retained measurable viscosity during the applied high-temperature test protocol. However, nanoparticle aggregation and sedimentation under concentrated acid and spent-acid conditions were not independently characterized.

#### 2.2.3. Dynamic Oscillatory Viscoelasticity and Plateau Modulus

**Figure 5:**
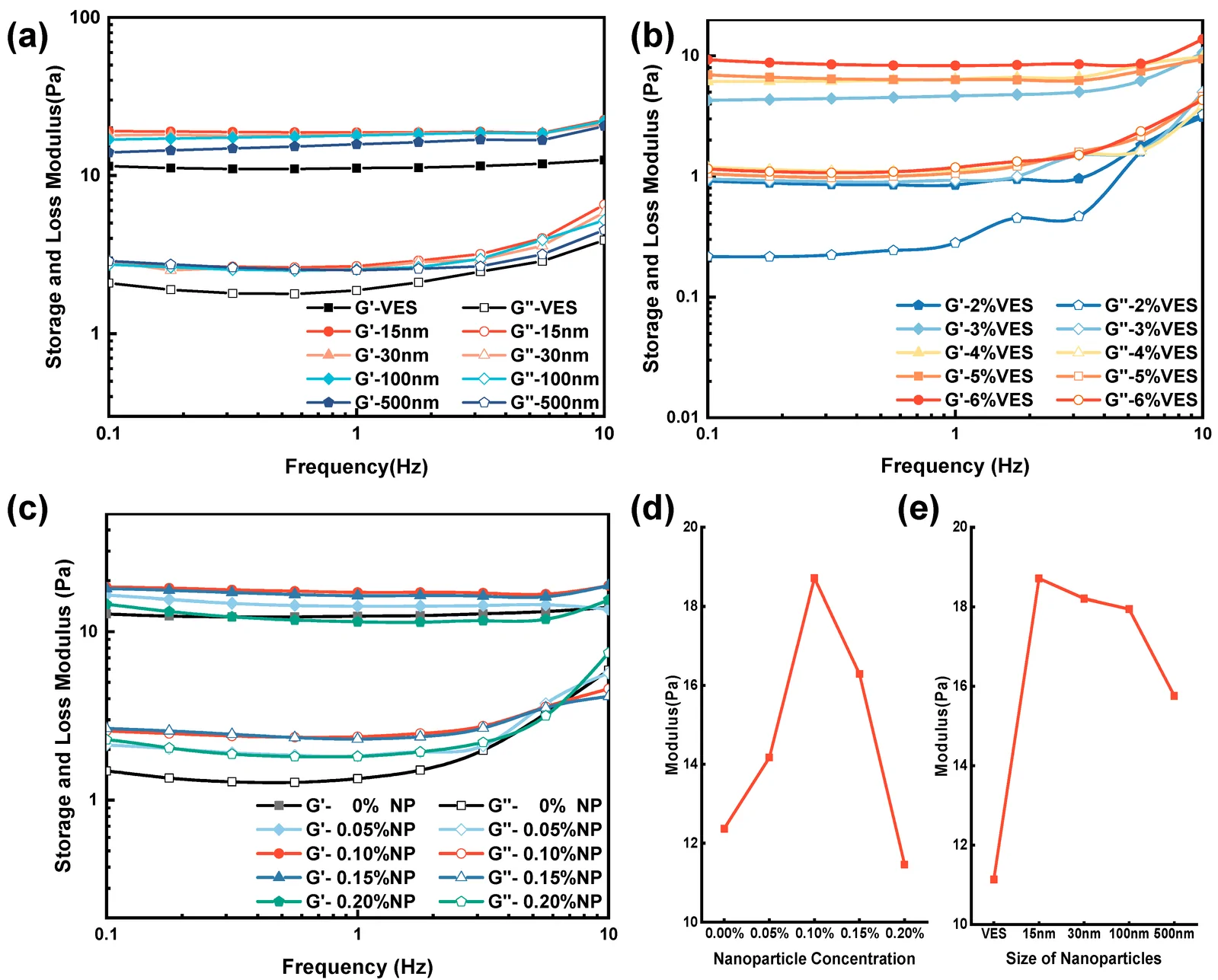
Frequency-dependent storage modulus G′ and loss modulus G″ of the VES and N-EVES systems at varying (**a**) nanoparticle sizes, (**b**) VES concentrations, and (**c**) nanoparticle concentrations; plateau storage modulus as a function of (**d**) nanoparticle concentration and (**e**) nanoparticle size. All formulations contained 15 wt% HCl, 6 wt% formulated VES solution, and 0.50 wt% corrosion inhibitor, except for the explicitly varied VES concentration, nanoparticle concentration, or nanoparticle size.

Dynamic oscillatory measurements were used to quantify the influence of nanoparticles on the viscoelastic properties of the system. Across the entire evaluated frequency range (0.1–10 Hz), both the storage modulus (G′) and loss modulus (G″) of all systems exhibited relatively flat plateau regions, with G′ consistently dominating G″ (Figure 5a–c). This behavior indicates a predominantly elastic response under the tested conditions. The incorporation of nanoparticles increases the viscoelastic moduli. Consistent with the steady-state shear viscosity observations, the concentration of 0.10 wt% yields the maximum G′ value (peaking at approximately 18.5 Pa), representing a substantial enhancement compared to the pristine VES system (G′ ≈ 11 Pa) (Figure 5d,e). The oscillatory results also show a particle-size-dependent rheological response. At an identical mass concentration, the 15 nm nanoparticles increase the storage modulus (G′) and loss modulus (G″) by 18% and 6%, respectively, compared to their 500 nm counterparts. This enhancement in elastic response is consistent with stronger nanoparticle-surfactant assochiation and increased micellar entanglement; however, direct micellar bridging was not structurally verified.

At an equal nanoparticle mass concentration, the observed rheological differences should not be attributed exclusively to particle diameter. For particles with comparable density and morphology, decreasing the nominal particle size increases both the particle number and the total available interfacial surface area. The 15 nm SiO_2_ nanoparticles may therefore provide more adsorption sites for surfactant molecules and more frequent nanoparticle-micelle contacts than the larger particles. Accordingly, their stronger reinforcement may reflect the combined effects of particle size, total surface area, particle number, and interfacial adsorption. Because the BET surface areas and aggregation states of the different nanoparticles were not independently measured, and no comparison was performed at an equal total surface area, these contributions cannot be quantitatively separated in the present study. The results are therefore interpreted as a size-dependent reinforcement effect under equal mass loading rather than definitive evidence of an intrinsic particle-size effect.

### 2.3. Zeta-Potential Analysis and Electrokinetic Interpretation

To explore possible nanoparticle-surfactant interactions, the electrokinetic properties of the diluted samples were investigated by zeta-potential analysis.

Because the high acidity and conductivity of the undiluted 15 wt% HCl formulation exceeded the recommended operating range of the instrument, all samples were diluted 100-fold before measurement. These measurements were used only for qualitative comparison. Such dilution substantially reduces ionic strength, expands the electrical double layer, and may alter micellar morphology and nanoparticle aggregation; therefore, the measured zeta potentials should not be regarded as representative of the original concentrated acid formulation. The positive potential of the VES may result from protonation of the carboxylate group of the betaine headgroup under acidic conditions. The near-zero potential observed for the 15 nm N-EVES system is consistent with charge neutralization, but it may also indicate an increased tendency toward aggregation. Accordingly, zeta potential data alone cannot confirm micellar bridging, pseudo-crosslinking, or colloidal stability.

**Figure 6:**
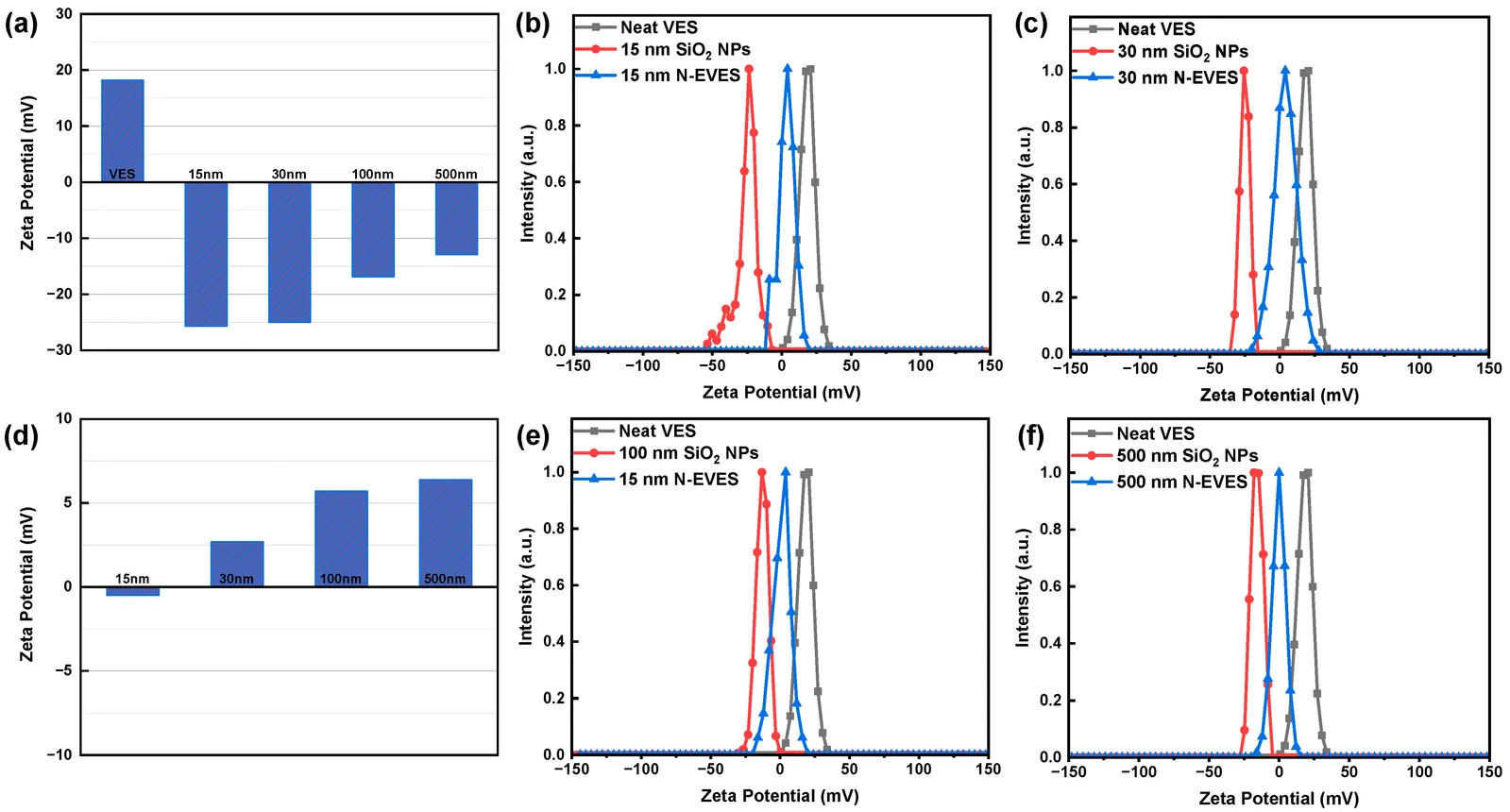
Zeta-potential measurements of the diluted VES, SiO_2_ nanoparticle, and N-EVES samples: (**a**) zeta potentials of the individual components; (**b**,**c**,**e**,**f**) corresponding potential-distribution profiles; and (**d**) particle-size-dependent zeta potentials of the mixed N-EVES samples. The intermediate values observed after mixing are consistent with nanoparticle-surfactant interactions under the diluted measurement conditions.

As illustrated in Figure 6a, the pristine C22^+^ VES system exhibits pronounced electropositivity (with a zeta potential of +18.2 mV), which may be associated with protonation of the betaine headgroup under acidic conditions. This positive potential indicates electrostatic repulsion among similarly charged aggregates under the diluted measurement conditions, although its direct relationship with high-temperature micellar stability was not independently established. Conversely, the pristine SiO_2_ nanoparticles (NPs) are universally electronegative in aqueous solutions, and the absolute values of their Zeta potentials demonstrate a striking size dependency: as the particle diameter decreases from 500 nm to 15 nm, the Zeta potential drops significantly from −13.0 mV to −25.8 mV. The more negative potential measured for the smaller-particle dispersions indicates a difference in their apparent electrokinetic surface properties under the diluted measurement conditions.

The potential-distribution profiles in Figure 6b,c,e,f show that the peaks of the mixed systems lie between those of the individual VES and nanoparticle samples. Across all N-EVES systems, the Zeta potential peaks of the mixtures consistently reside between those of the pure VES and the pristine NPs, which is consistent with electrostatic interaction and adsorption between the VES components and SiO_2_ nanoparticles.

The mixed systems also exhibited particle-size-dependent zeta potentials (Figure 6d). Under strongly acidic conditions, protonation of the carboxylate group reduces its negative charge, while the quaternary ammonium group remains positively charged, resulting in an overall positive electrokinetic potential. For the 500 nm N-EVES system, the system retains relatively high electropositivity (+6.4 mV) upon their incorporation; thus, inter-micellar electrostatic repulsion persists. However, when the ultra-small 15 nm nanoparticles are employed, the Zeta potential of the N-EVES system is drastically neutralized to a near-zero state (−0.5 mV).

The shift toward a near-zero zeta potential may reflect charge neutralization associated with nanoparticle-surfactant adsorption. The larger estimated interfacial area of the 15 nm nanoparticles may provide more potential adsorption sites and promote closer association between nanoparticles and VES aggregates. However, a near-zero zeta potential may also indicate an increased tendency toward aggregation. Because the measurements were performed after 100-fold dilution, they cannot establish electrical-double-layer compression, micellar bridging, or colloidal stability in the original 15 wt% HCl formulation. From an electrokinetic perspective, the measured trends are nevertheless consistent with the stronger rheological response observed for the 15 nm nanoparticles.

### 2.4. Proposed Mechanism of Nanoparticle-Mediated Micellar Stabilization

**Figure 7:**
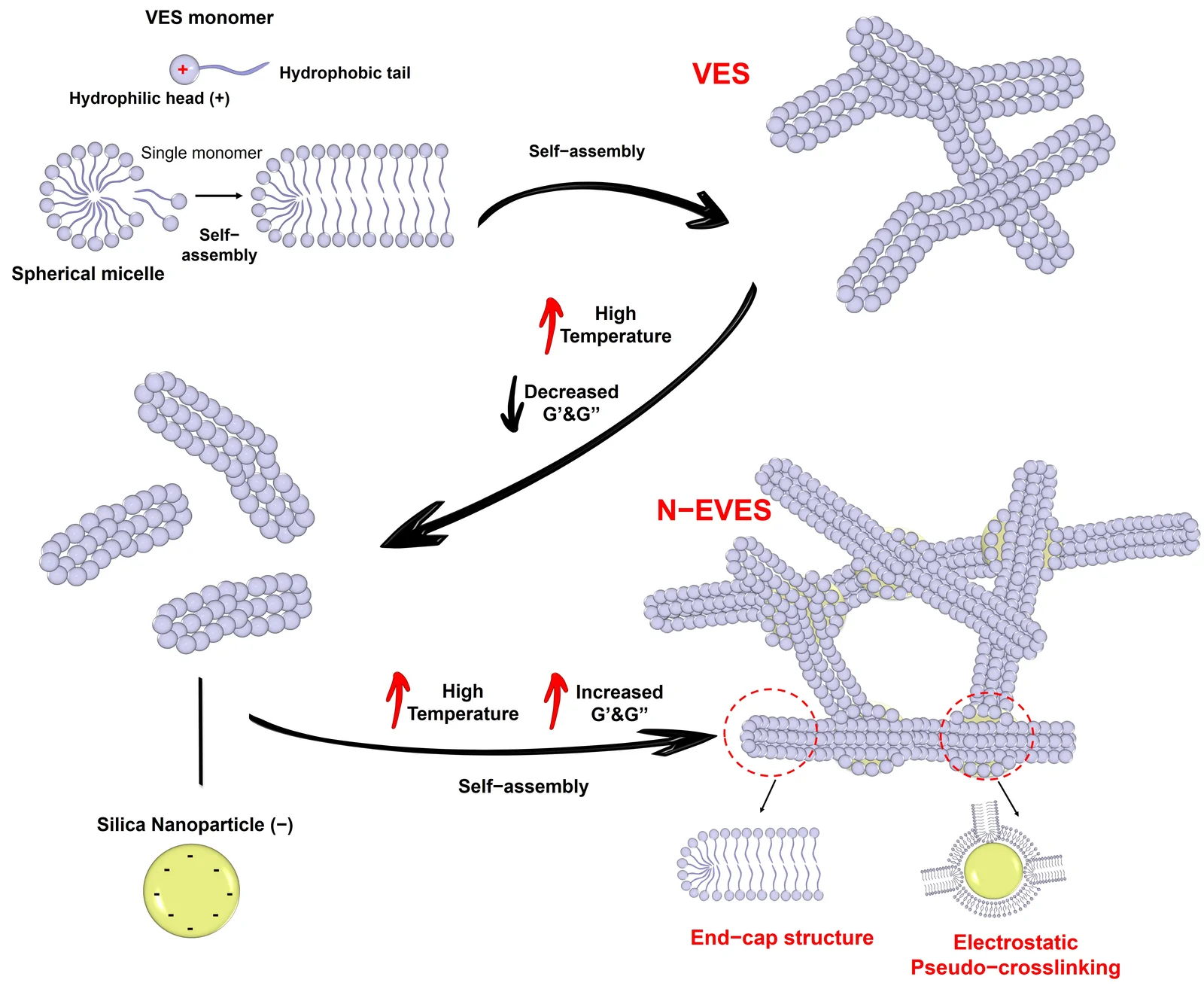
Proposed schematic mechanism of nanoparticle-mediated stabilization of the N-EVES system, including possible pseudo-crosslinking and end-capping interactions. These structures remain hypothetical and require direct structural verification.

Integrating the aforementioned rheological and zeta-potential analyses, this study proposes a nanoparticle-mediated microstructural stabilization mechanism for the N-EVES system at elevated temperatures (illustrated in Figure 7). In conventional systems, VES monomers with zwitterionic hydrophilic headgroups self-assemble into wormlike micelles (WLMs) in aqueous solutions. However, under high-temperature conditions, intense thermal motion disrupts the hydrophobic association equilibrium, leading to the thermal scission of micelles and a subsequent drastic attenuation of the viscoelastic moduli (G′ and G″).

Upon introducing SiO_2_ nanoparticles with highly electronegative surfaces, intense self-assembly is triggered between the electropositive VES micellar headgroups and the anionic nanoparticle surfaces, driven by electrostatic attraction. As highlighted in the bottom-right inset of Figure 7, two possible nanoparticle-mediated interactions are proposed. First, the nanoparticles may interact with micellar termini and form proposed end-cap-like structures which could reduce the accessibility or reactivity of micellar end regions. Second, acting as versatile “Electrostatic Pseudo-crosslinking Centers,” a single nanoparticle may adsorb VES aggregates at multiple surface sites and thereby promote inter-micellar association. Specifically, the high interfacial area associated with the 15 nm nanoparticles may promote the formation of a more strongly entangled micellar network. This proposed mechanism provides a possible interpretation of the enhanced rheological performance of the N-EVES system.

### 2.5. Acid-Rock Reaction Kinetics and Retardation

#### 2.5.1. Evolution of Surface Reaction Rate and Effective Mass Transfer Characteristics

To further evaluate the retarded acidizing potential of the N-EVES system in deep carbonate reservoirs, the acid-rock reaction kinetics were investigated using a high-temperature and high-pressure (HTHP) rotating disk apparatus. Carbonate dissolution behavior is governed by the competition among intrinsic surface reaction, reactant transport, and fluid convection. Changes in this balance can produce different dissolution regimes, including face dissolution, dominant wormholing, and more spatially distributed dissolution [25,26].

**Figure 8:**
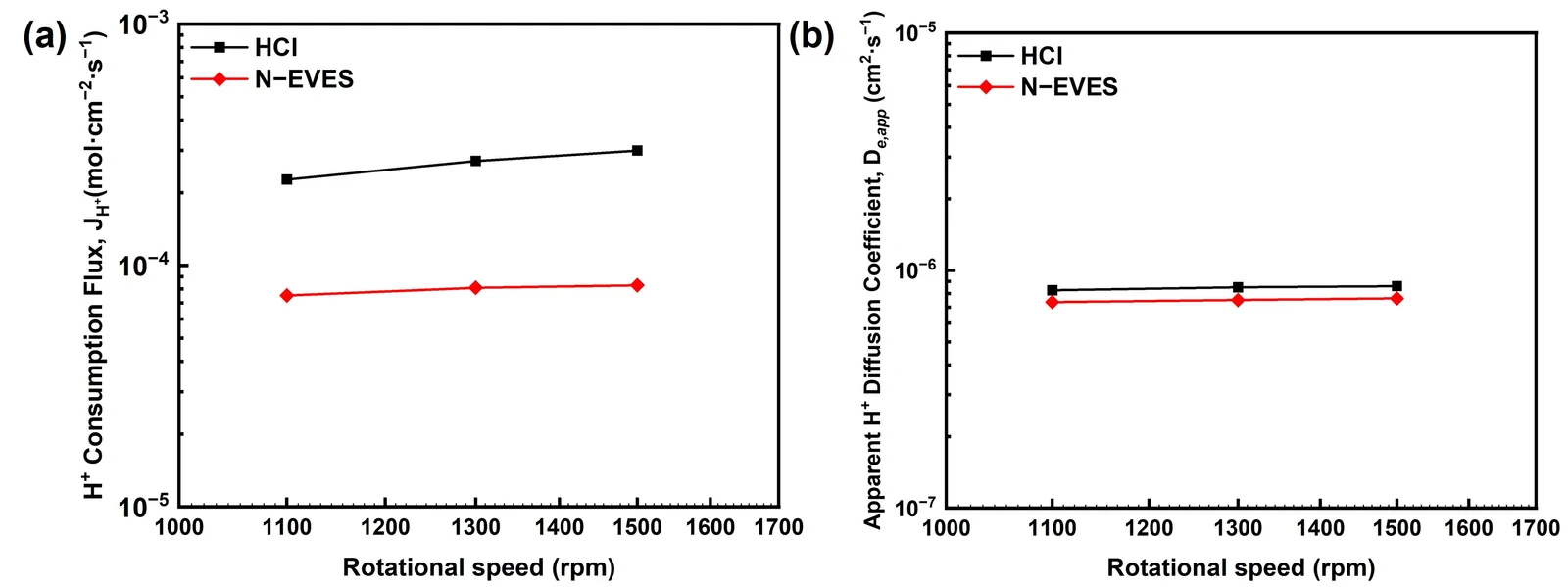
Rotating disk acid-rock reaction results for conventional HCl and the N-EVES formulation at 160 °C: (**a**) area normalized H^+^ consumption flux and (**b**) apparent effective H^+^ diffusion coefficient. Conditions: 500 mL acid, 5.07 cm^2^ exposed area, 30 min, and 1100–1500 rpm.

The experimental results show differences in the area-normalized H^+^ consumption flux and apparent H^+^ transport behavior between conventional HCl and the N-EVES system. The weak dependence of the reaction rate on rotational speed in Figure 8 suggests a mixed kinetic regime at 160 °C rather than a purely mass-transfer-controlled process.

Within 1100–1500 rpm, the H^+^ consumption fluxes of both systems increased only slightly with rotational speed. This weak dependence indicates that both interfacial reaction and proton transport contributed under the tested conditions. At identical speeds, the N-EVES flux was markedly lower than that of conventional HCl. The HCl flux ranged from 2.0 × 10^−4^ to 3.0 × 10^−4^ mol·cm^−2^·s^−1^, whereas the N-EVES flux remained below 1.0 × 10^−4^ mol·cm^−2^·s^−1^.

Under the tested conditions, the N-EVES value was approximately 25% of that measured for conventional HCl. This reduction may be associated with increased bulk viscosity and the surface adsorption or deposition observed by SEM-EDS. The higher bulk viscosity may increase transport resistance near the solid–liquid interface;; however, the local boundary layer viscosity was not directly measured. Accordingly, the reduction is discussed as a combined bulk rheological and interfacial effect, without assigning the relative contribution of either factor.

Figure 8b presents the apparent effective H^+^ diffusion coefficient estimated using the adopted rotating disk relationship. The N-EVES and conventional HCl values were approximately 8.0 × 10^−7^ and 9.0 × 10^−7^ cm^2^·s^−1^, respectively, and thus remained within the same order of magnitude under the tested conditions. Because the relationship is applied to a rheologically complex fluid, the coefficients are interpreted as apparent comparative transport parameters rather than intrinsic molecular diffusivities. The weak rotational dependence indicates that both interfacial reaction and proton transport may contribute [26,27].

#### 2.5.2. Microscopic Evolution of the Acid-Rock Interface and Surface Deposition

To examine the surface changes associated with the lower reaction rate of the N-EVES system, the rock-surface morphologies and elemental compositions after different treatments were analyzed using SEM-EDS.

**Figure 9:**
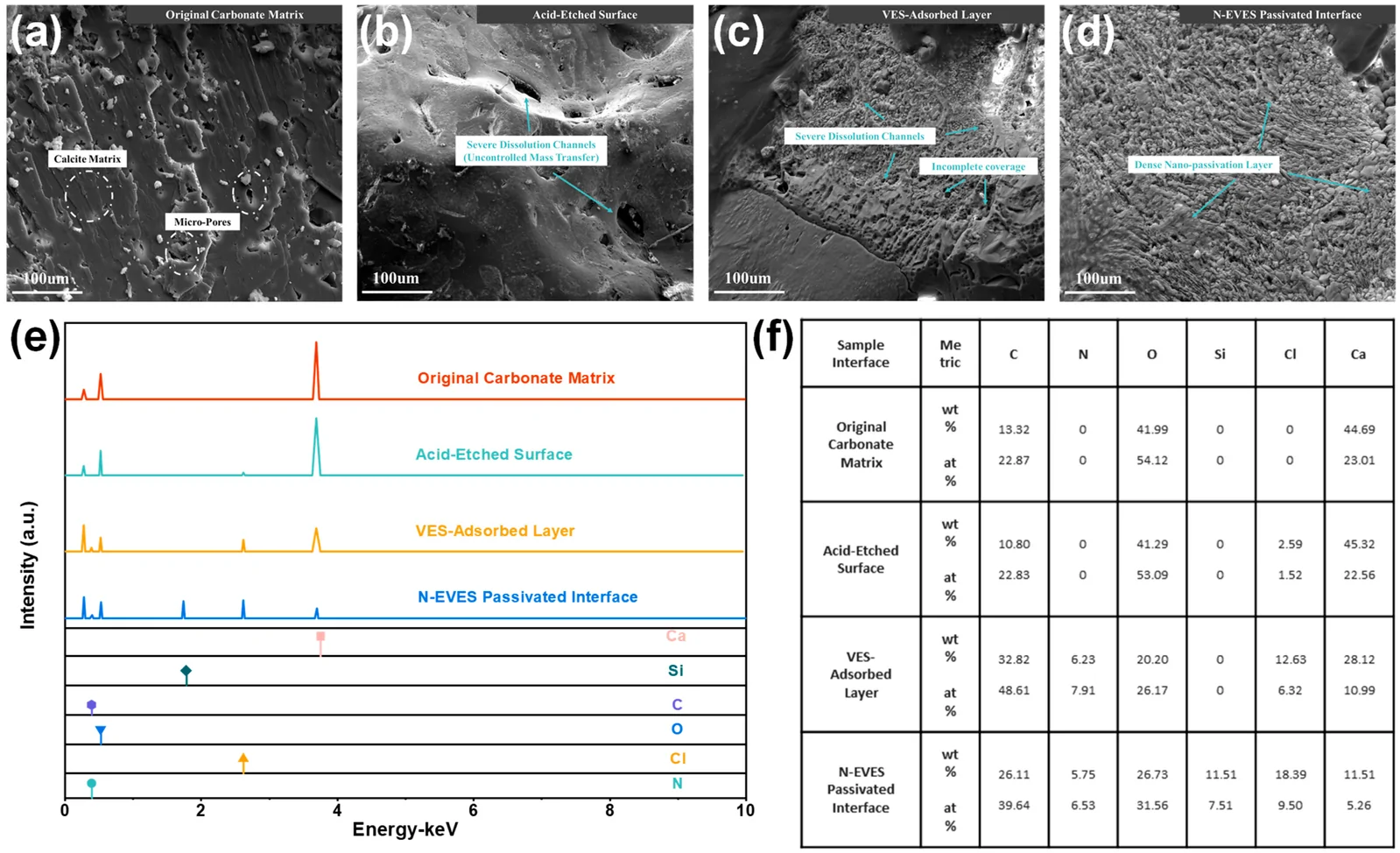
Surface morphology and elemental composition after different acid treatments: SEM micrographs of (**a**) the original carbonate matrix, (**b**) the HCl-treated surface, (**c**) the VES-treated surface, and (**d**) the N-EVES-treated surface; (**e**) corresponding EDS spectra and (**f**) elemental compositions (wt% and at%). The Si and N signals support surface adsorption or deposition after N-EVES treatment.

The SEM images in Figure 9a–d show distinct surface morphologies after the different treatments. The pristine carbonate matrix exhibits a relatively smooth surface (Figure 9a), whereas the HCl-treated surface shows pronounced dissolution features (Figure 9b). Surface deposits together with localized dissolution are observed after VES treatment (Figure 9c). The N-EVES-treated surface exhibits more extensive surface coverage by deposited material (Figure 9d); however, the SEM images alone do not establish the continuity or thickness of this deposit.

The EDS spectra and elemental compositions in Figure 9e,f provide additional information on the surface deposits. Compared with the pristine rock, the N-EVES-treated surface contains detectable Si and N signals, with measured contents of up to 26 at% and 6 at%, respectively. These results support the adsorption or deposition of SiO_2_ nanoparticles and VES-derived species on the carbonate surface. Combined with the lower measured reaction rate, the SEM-EDS observations suggest that this surface deposit may contribute to interfacial reaction retardation. Its continuity, compactness, and thickness were not directly determined.

### 2.6. Single-Core Flooding and Dynamic Flow Resistance

#### 2.6.1. Evolution of Dynamic Flow Resistance and Breakthrough Behavior

To evaluate the dynamic flow resistance and diversion behavior of the N-EVES system within formation porous media, single-core flooding experiments were conducted on carbonate cores with varying permeability levels at 160 °C. The dynamic core-flooding performance was evaluated using the dimensionless pressure-drop ratio (PDR), maximum pressure-drop ratio (MPDR), and pore volume to breakthrough (PVbt), as defined in Section 4.6. The PDR was recorded as a function of injected pore volume, and PVbt was determined for nanoparticle concentrations ranging from 0.025 to 0.20 wt%.

1. Permeability-Dependent Flow Resistance

**Figure 10:**
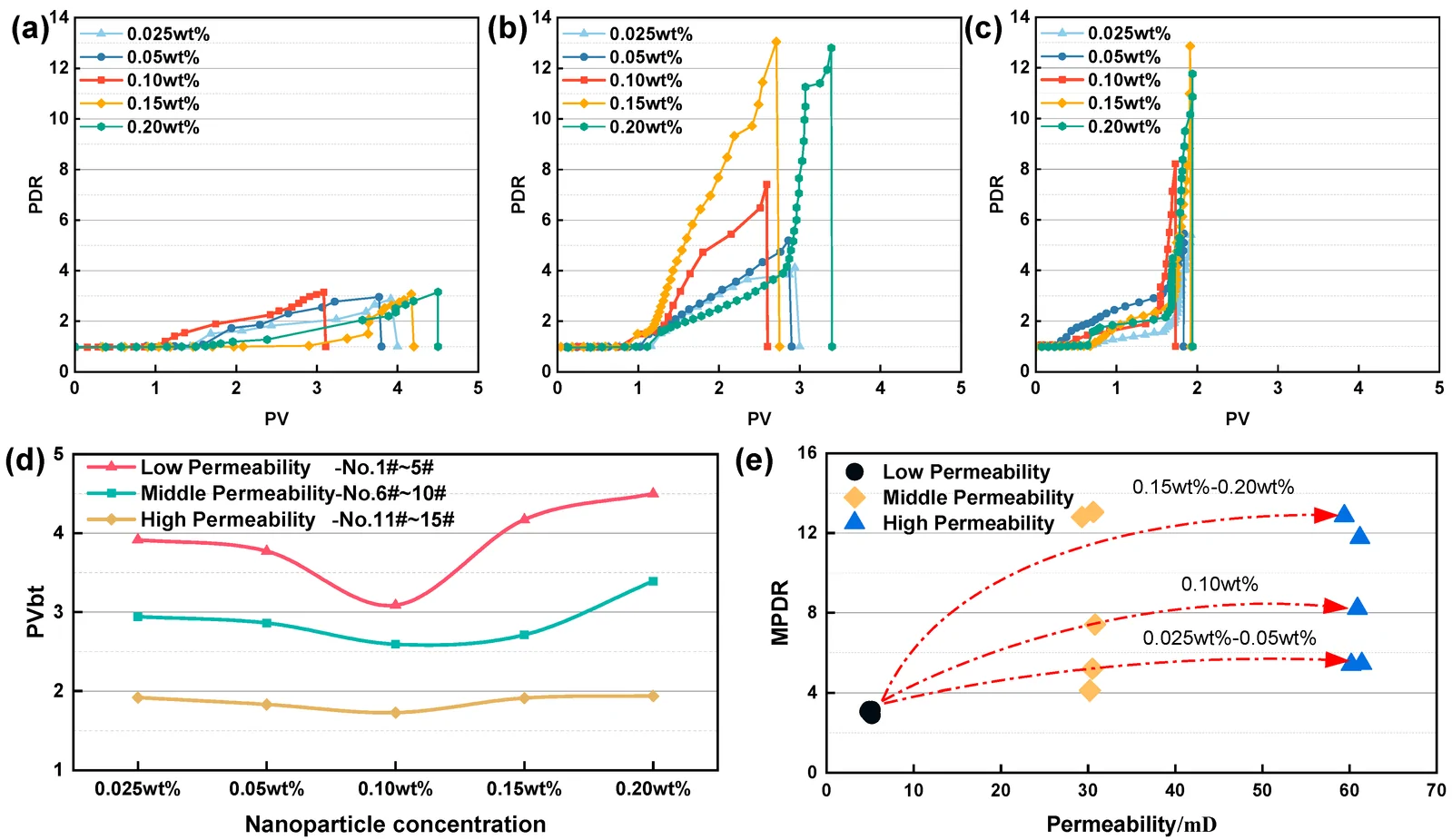
Dynamic flow-resistance and breakthrough evaluation of the N-EVES systems via single-core flooding tests at 160 °C: evolution of pressure drop ratio (PDR) in (**a**) low, (**b**) middle, and (**c**) high permeability cores; (**d**) breakthrough pore volume (PVbt) as a function of nanoparticle concentration showing the concentration-dependent breakthrough behavior; and (**e**) relationship between maximum PDR and rock permeability demonstrating the permeability-dependent flow resistance. Cores No. 1–5, No. 6–10, and No. 11–15 correspond to the low-, medium-, and high-permeability groups, respectively.

Based on the PDR evolution curves for different core permeabilities (Figure 10a–c) and the relationship between MPDR and permeability (Figure 10e), the N-EVES system exhibited permeability-dependent flow resistance. In low-permeability cores (~5 mD), the MPDR remained approximately 3.0, whereas in high-permeability cores (~60 mD), the MPDR reached approximately 8.0 and, in some cases, exceeded 12.0 at nanoparticle concentrations of 0.10 wt% and above. These pressure responses demonstrate increased flow resistance in the high-permeability cores and could promote subsequent flow redistribution. However, they do not independently identify the micellar state formed after acid spending.

2. Effect of Nanoparticle Concentration on Etching Efficiency

The pore volume to breakthrough (PV_bt_) is a measure of the acid volume required to achieve wormhole breakthrough. As illustrated by the PVbt-nanoparticle concentration profiles in Figure 10d, cores No. 1–5, No. 6–10, and No. 11–15 represent the low-, medium-, and high-permeability groups, respectively. The corresponding nanoparticle concentrations ranged from 0.025 to 0.20 wt%. As the nanoparticle concentration increased from 0.025 wt% to 0.10 wt%, the PV_bt_ of the low-permeability cores decreased to a minimum of approximately 3.0 PV. When the concentration was further increased to 0.20 wt%, PVbt increased to approximately 4.5 PV. The minimum PVbt observed at 0.10 wt% is consistent with the higher rheological response measured at this concentration. Among the tested formulations, the 0.10 wt% N-EVES system provided the most favorable balance between flow resistance and acid penetration in the low-permeability cores.

#### 2.6.2. Three-Dimensional CT Visualization of Post-Treatment Wormhole Morphology

**Figure 11:**
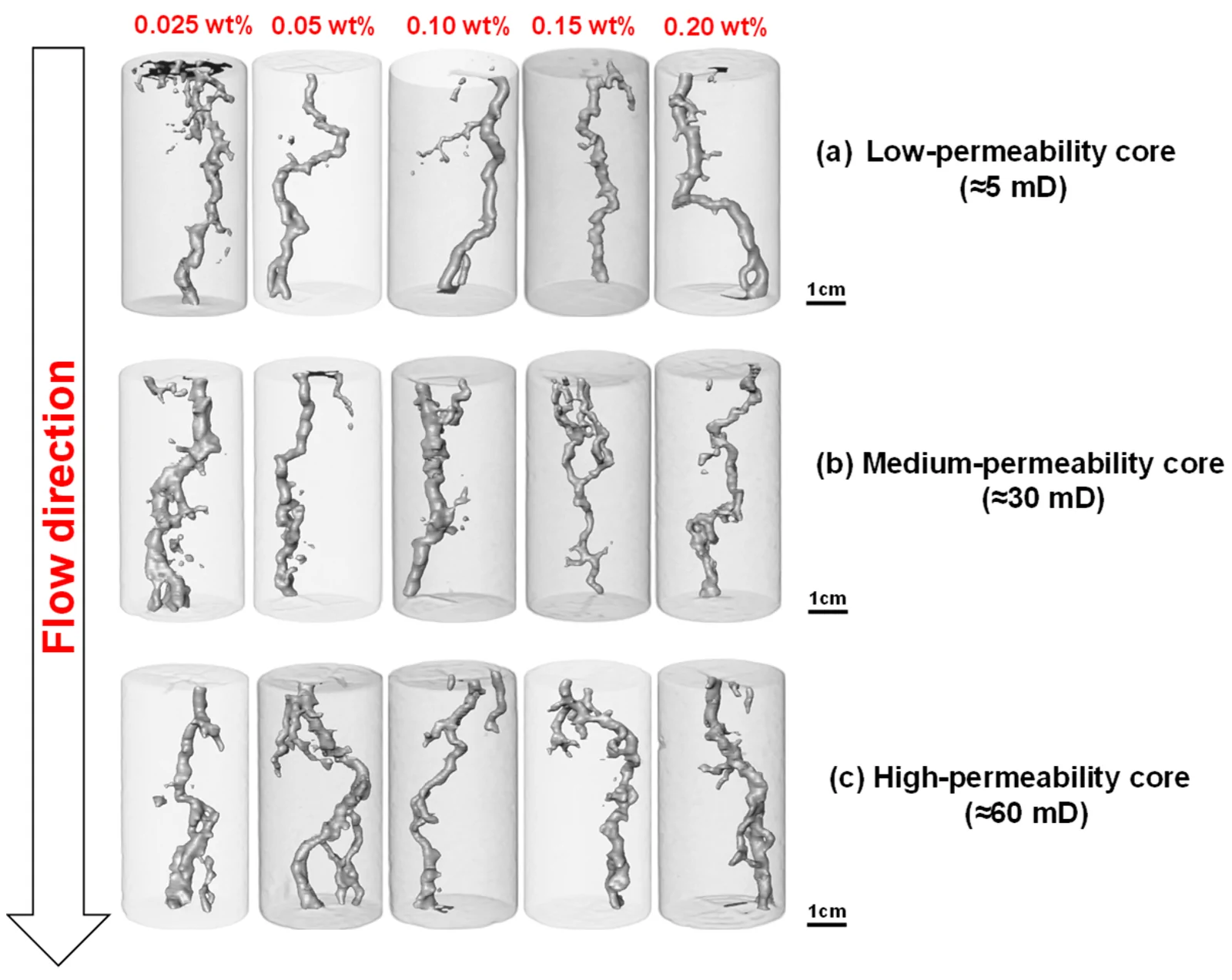
3D-CT reconstructed wormhole morphologies illustrating the selective plugging and intelligent diversion behavior of the N-EVES systems: morphological evolution in (**a**) low (~5 mD), (**b**) medium (~30 mD), and (**c**) high (~60 mD) permeability cores across varying nanoparticle concentrations, showing a dominant wormhole morphology at 0.10 wt% among the tested concentrations. The arrows indicate the flow direction from the inlet to the outlet. The scale bar represents 1 cm.

To compare post-treatment dissolution patterns, micro-CT scanning and three-dimensional wormhole reconstruction were performed on the carbonate cores after single-core flooding. Previous continuum- and pore-scale studies have shown that wormhole initiation, branching, and breakthrough are controlled by coupled flow, transport, and reaction processes, and are sensitive to local permeability heterogeneity and flow focusing [28,29,30,31]. The evolution of wormhole morphology across varying permeabilities and nanoparticle concentrations is illustrated in Figure 11. These 3D architectures provide rigorous mutual verification with the macroscopic fluid responses, specifically the pressure drop ratio (PDR) and pore volume to breakthrough (PV_bt_), discussed previously.

1. Dominant Wormholes and Concentration-Dependent Penetration in Low-Permeability Matrices

In the low-permeability (~5 mD, Figure 11a) and medium-permeability (~30 mD, Figure 11b) cores, the observed wormhole morphologies are consistent with the non-monotonic variation of PVbt. At a low nanoparticle concentration (0.025 wt%), significant face dissolution occurs at the core inlet, resulting in broad wormholes with numerous ineffective branches, which consume excessive acid (corresponding to a higher PV_bt_). In contrast, at 0.10 wt%, the acid etched a relatively straight, slender, and single dominant wormhole within the low-permeability medium. This morphology corresponded to the minimum PVbt observed among the tested concentrations. At an excessive concentration (0.20 wt%), the wormhole morphology became more tortuous and branched. This pattern may be associated with increased transport resistance, although the underlying mechanism was not directly determined.

2. Branched Wormhole Morphologies in High-Permeability Cores

Within the high-permeability cores (~60 mD, Figure 11c), the N-EVES system manifests a starkly different 3D etching topography. At a nanoparticle concentration of 0.10 wt%, the wormholes do not form a single dominant channel but instead exhibit a highly complex, curved, and branched network. The branched morphology observed at 0.10 wt% is consistent with the higher MPDR measured in the corresponding high-permeability core. The elevated pressure response indicates increased local flow resistance within the high-permeability core. This increased resistance may have promoted redistribution of the injected fluid toward other available flow paths, resulting in a more branched dissolution morphology. However, the post-treatment CT images do not directly demonstrate in situ gel-slug formation or resolve the time-dependent redistribution process.

The observed variation from relatively localized channels to more branched dissolution patterns is broadly consistent with the established sensitivity of reactive dissolution to permeability heterogeneity and local flow redistribution [28,29,30,31]. However, the present CT images provide a post-treatment morphological comparison and do not independently resolve the time-dependent development of individual wormholes.

### 2.7. Flow Redistribution and Wormhole Morphology Under a High Permeability Contrast

**Figure 12:**
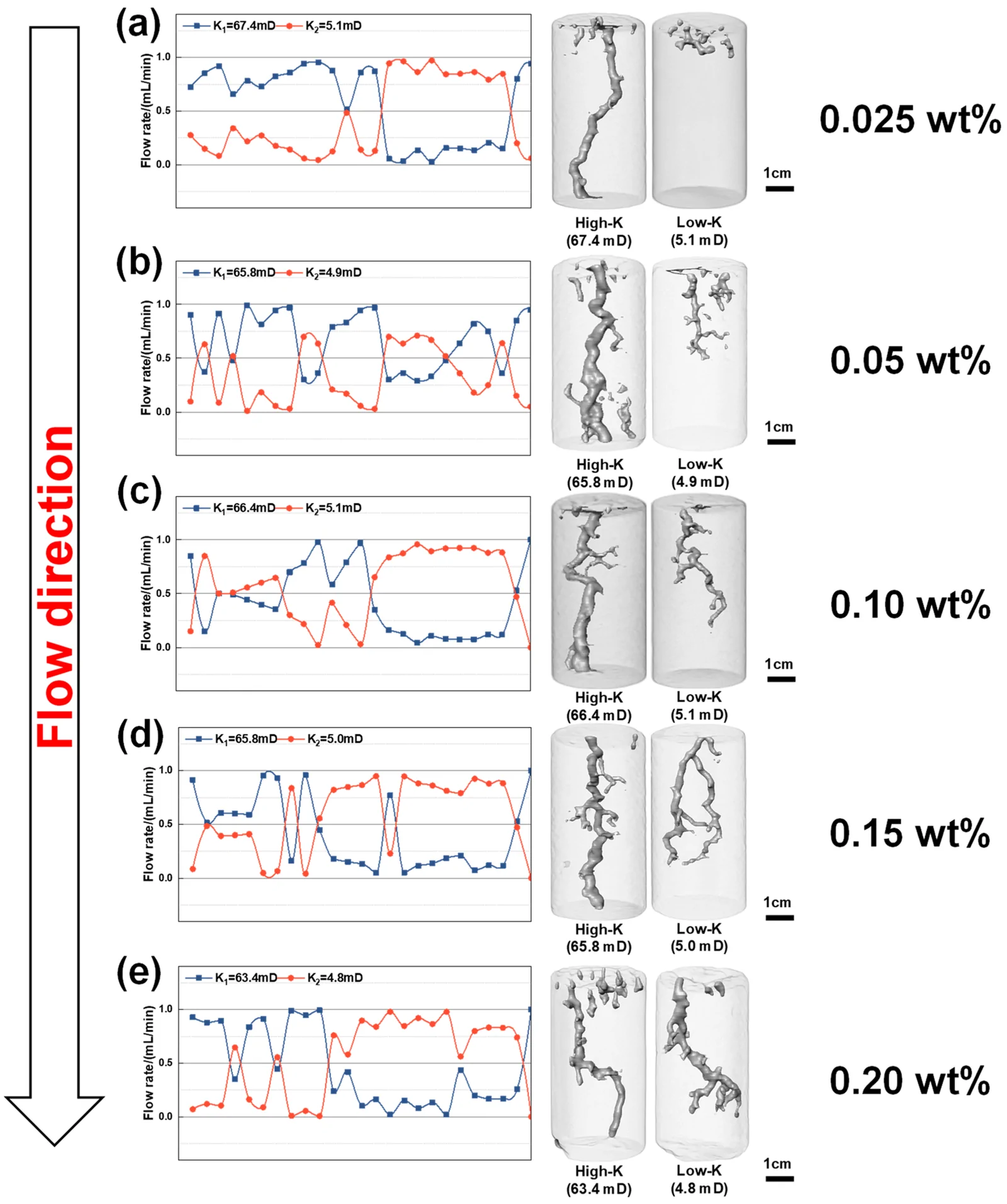
Dynamic fluid diversion and 3D wormhole evolution of the N-EVES system in parallel dual-core flooding at 160 °C. (**a**–**e**) Flow rate distributions and corresponding CT reconstructions of high- and low-permeability cores at nanoparticle concentrations from 0.025 to 0.20 wt%. Note: All subfigures share the common horizontal axis shown below the bottom subplot. The high- and low-permeability core curves are identified in each panel. The abrupt changes and oscillations reflect transient flow redistribution between the two parallel branches as their relative flow resistances evolve during acid-rock reaction. Continuous injection was maintained without valve switching or pump shutdown.

To evaluate the flow-redistribution behavior of the N-EVES system under a high permeability contrast, a parallel dual-core physical model was constructed with a permeability contrast of approximately 13 (High-K ≈ 65 mD, Low-K ≈ 5 mD). Figure 12 presents the flow-rate profiles and post-treatment CT reconstructions for the high- and low-permeability cores. The flow-rate and CT results were compared to assess the consistency between fluid allocation and the resulting dissolution morphologies. Preferential flow through the higher-permeability branch is expected in heterogeneous systems, whereas changes in branch resistance can redistribute the injected fluid and consequently alter the resulting dissolution patterns [30,31].

1. Low Concentration Regime (0.025–0.05 wt%)

At 0.025 wt%, the measured flow resistance was insufficient to produce substantial redistribution toward the low-permeability branch. Throughout the flooding process, acid predominantly channels through the high-permeability core (blue curve), while the flow rate in the low-permeability core (red curve) remains low. CT reconstructions corroborate this: a broad, dominant wormhole is etched in the high-permeability core, whereas the low-permeability core exhibited mainly inlet-face dissolution, consistent with limited acid penetration. At 0.05 wt%, the low-permeability flow rate increased slightly, although the flow curves exhibited pronounced oscillations. The corresponding CT images reveal that the wormhole in the high-permeability core remains broad, and the diversion effect is highly unstable.

2. The 0.10 wt% Regime: Enhanced Flow Redistribution

At 0.10 wt%, the system showed the strongest flow redistribution among the tested concentrations. During the initial 10 min of flooding, acid preferentially entered the high-permeability channels. However, as the acid-rock reaction progressed, the hydraulic resistance of the high-permeability branch increased, causing the injected fluid to redistribute toward the low-permeability branch. This behavior is consistent with possible in situ viscosity buildup after acid spending, but does not directly demonstrate micellar gel formation. The high-permeability flow rate decreased sharply at the 12 min mark and stabilized below 0.2 mL/min, after which the subsequently injected acid is successfully diverted into the low-permeability matrix, leading to a significant surge in the low-permeability flow rate (red curve). The corresponding CT reconstructions are consistent with this flow redistribution: the high-permeability core contained a shorter, more branched dissolution network, whereas the low-permeability core exhibited a more extended dominant wormhole. Among the tested concentrations, the 0.10 wt% N-EVES system produced the strongest flow redistribution toward the low-permeability core.

3. High Concentration Regime (0.15–0.20 wt%): Over-crosslinking and Flow Oscillations

Further increasing the concentration to 0.15 wt% and 0.20 wt% (Figure 12d,e) continued to increase fluid allocation toward the low-permeability core; however, the higher nanoparticle concentrations produced stronger and more variable flow resistance. Macroscopically, this is manifested as high-frequency flow oscillations between the high- and low-permeability cores. These oscillations indicate repeated changes in the relative hydraulic resistance of the two branches, although their specific microscopic origin cannot be determined from the flow-rate curves alone.

The broad and branched wormholes observed in both cores may be associated with less localized acid transport and more spatially distributed dissolution at the higher nanoparticle concentrations. These conditions produced less favorable axial penetration patterns than the 0.10 wt% formulation.

Based on the segmented CT reconstruction, the normalized axial wormhole penetration fraction in the low-permeability core treated with the 0.10 wt% formulation was approximately 70%. This value represents the ratio of axial wormhole penetration length to total core length and does not represent a 70% improvement relative to a control or volumetric sweep efficiency.

Furthermore, contact with produced hydrocarbons or dilution during flowback may reduce the viscosity of the VES-based network and facilitate post-treatment fluid recovery. The post-flush permeability recovery and residual retention of VES or nanoparticles were not evaluated in the present study.

### 2.8. Consistency Among Complementary Experimental Findings

The main experimental findings were evaluated using complementary characterization and performance measurements. The higher apparent viscosity of the N-EVES system was accompanied by higher storage and loss moduli, including a higher plateau, consistently indicating enhanced rheological strength under the applied test conditions. The reduced acid-rock reaction rate was evaluated together with the Si- and N-containing species detected on the treated carbonate surface, which is consistent with possible contributions of bulk fluid resistance and surface adsorption or deposition. In the parallel dual-core experiments, the pressure response and increased low-permeability outlet-flow fraction were consistent with the more spatially extended wormhole observed by 3D-CT in the low-permeability core. The overall consistency among the rheological, kinetic, microscopic, and core-flooding observations supports the reported macroscopic performance trends. However, this cross-validation does not directly verify nanoparticle bridging, electrostatic pseudo-crosslinking, micellar topological reconstruction, or end-cap-like structures at the molecular level.

## 3. Conclusions

In this study, silica-nanoparticle reinforcement of a long-chain C22^+^ wormlike micellar acid system was evaluated under high-temperature carbonate-acidizing conditions. Building on the established literature concerning nanoparticle-enhanced WLM systems, the present work examined the relationships among nanoparticle-size- and concentration-dependent rheological behavior, acid-rock reaction kinetics, surface adsorption or deposition, and flow redistribution in heterogeneous carbonate cores. The principal findings under the applied experimental conditions are summarized as follows:

### 3.1. Experimentally Demonstrated Findings

1. Under the applied temperature-ramp protocol, the N-EVES system retained higher apparent viscosity and viscoelastic moduli than the corresponding VES system during the 20 min isothermal holding period at 160 °C. Among the tested formulations, the formulation containing 0.10 wt% of 15 nm SiO_2_ nanoparticles exhibited the highest measured rheological response, with an apparent viscosity of approximately 210 mPa·s and a plateau storage modulus of approximately 18.5 Pa, compared with a plateau storage modulus of approximately 11 Pa for the corresponding VES system without nanoparticles.
2. At equal nanoparticle mass concentration, the 15 nm nanoparticles produced G′ and G″ values that were approximately 18% and 6% higher, respectively, than those obtained with the 500 nm nanoparticles, indicating a particle-size-dependent rheological response under the tested conditions.
3. The N-EVES formulation reduced the acid-rock reaction rate to approximately 25% of that measured for conventional HCl under the tested conditions. The apparent effective H^+^ diffusion coefficients of the N-EVES and HCl systems were approximately $8.0\times 10^{-7}$ and $9.0\times 10^{-7}\;cm^{2}\;s^{-1}$, respectively. SEM-EDS analysis detected Si- and N-containing species on the N-EVES-treated carbonate surface, consistent with surfactant- and nanoparticle-associated surface adsorption or deposition.
4. Parallel dual-core flooding under a permeability contrast of approximately 13 showed flow redistribution toward the low-permeability core. Among the tested formulations, the 0.10 wt% nanoparticle formulation produced the strongest measured redistribution toward the low-permeability branch. The corresponding CT reconstruction showed a normalized axial wormhole penetration fraction of approximately 70% in the low-permeability core.

### 3.2. Interpretations Indirectly Supported by the Data

The rheological, zeta-potential, and microscopic results suggest that nanoparticle-surfactant association may modify interfacial charge characteristics and promote micellar association or entanglement, thereby contributing to the enhanced rheological response. The lower reaction rate and the detected Si- and N-containing surface deposits further suggest that increased fluid resistance and surface adsorption or deposition may jointly contribute to acid-rock reaction retardation. However, the relative contributions of these effects were not independently quantified.

### 3.3. Proposed Mechanisms and Study Limitations

Direct nanoparticle bridging, electrostatic pseudo-crosslinking, micellar end-capping, and topological reconstruction remain proposed molecular-level mechanisms requiring direct structural verification under representative acidic and high-temperature conditions. The present experiments also do not establish long-duration isothermal stability at 160 °C. Long-term thermal behavior, nanoparticle stability in concentrated and spent acid, reservoir-brine compatibility, post-treatment cleanup, corrosion performance, and permeability retention were not evaluated and require further investigation.

### 3.4. Practical Implications

The enhanced viscosity retention, reduced acid-rock reaction rate, and improved flow redistribution observed under the tested conditions indicate that the N-EVES system may have potential to improve acid placement in high-temperature, heterogeneous carbonate reservoirs. The formulation may help retard rapid acid consumption in high-permeability pathways and promote acid penetration into lower-permeability regions. The present findings extend previous nanoparticle-WLM research to a strongly acidic, high-temperature carbonate-acidizing environment and provide application-specific guidance for formulation screening and performance evaluation. Further reservoir-specific evaluation of corrosion control, brine compatibility, cleanup performance, injectivity, nanoparticle retention, and field-scale applicability is required before practical deployment.

## 4. Materials and Methods

### 4.1. Materials

**Figure 13:**
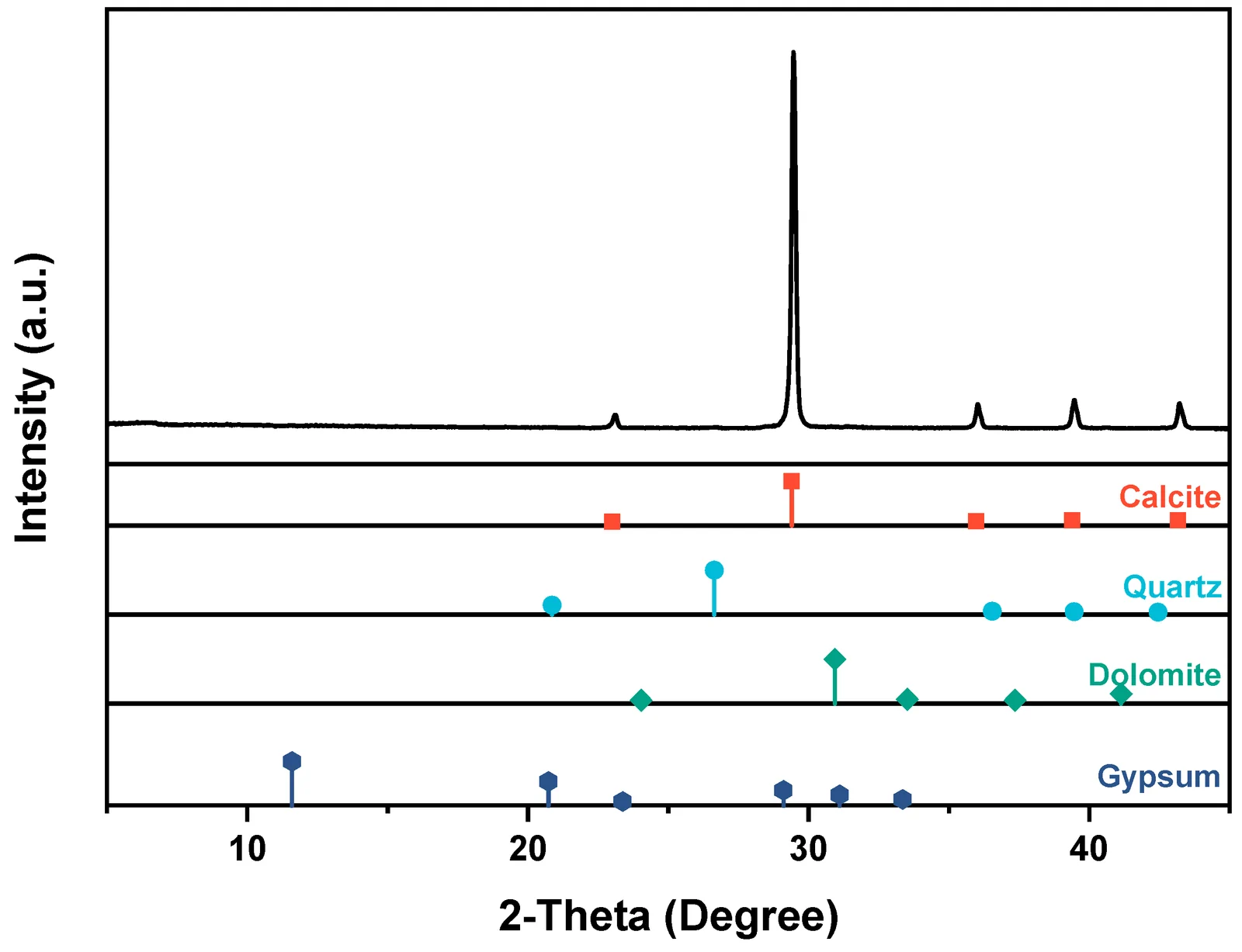
X-ray diffraction (XRD) pattern and mineralogical phase identification of the Indiana carbonate core, showing calcite as the dominant crystalline phase.

1. VES: The C22^+^ VES used in this study was synthesized in-house and belongs to the erucamidopropyl betaine family of long-chain zwitterionic surfactants. The synthesized product was formulated as an aqueous solution containing approximately 40 wt% active matter. Owing to intellectual-property and confidentiality restrictions, its exact chemical name, molecular structure, detailed synthesis and purification procedures, and complete spectroscopic characterization are not disclosed. Unless otherwise specified, the reported VES concentrations of 2–6 wt% refer to the formulated solution, corresponding to approximately 0.8–2.4 wt% active surfactant. The absence of complete molecular and synthetic information represents a limitation with respect to independent molecular-level reproduction of the surfactant.
2. Nanoparticles: Commercial hydrophilic, approximately spherical silica nanoparticles (SiO_2_ NPs) with nominal average diameters of 15, 30, 100, and 500 nm were supplied by Aladdin (Riverside, CA, USA). The stated purity of the nanoparticles was 99.5%. The four nominal particle sizes were investigated at equal mass loading to evaluate their particle-size-dependent effects on the rheological behavior of the VES system.
3. Acid Solutions, Corrosion Inhibitor, and Water: The experimental acid solutions were prepared using 37% concentrated hydrochloric acid (HCl) as the stock solution, subsequently diluted with deionized (DI) water to the required concentrations. The high-temperature corrosion inhibitor was supplied by Komashi (Beijing, China). All solutions were formulated using DI water to minimize uncontrolled ionic effects and isolate the contributions of the VES and SiO_2_ nanoparticles. Therefore, the present experiments were not intended as a comprehensive evaluation of compatibility with reservoir brines of different salinity and hardness.
4. Rock Samples: Natural high-purity Indiana limestone, consisting predominantly of calcite (>99%), was used for all core-flooding experiments (Figure 13). The cores were 2.5 cm in diameter and 5.0 cm in length, with porosities of 12–15% and measured gas permeabilities of 1–100 mD. After cleaning and oven-drying, the porosity and gas permeability of each core were measured.

Unless otherwise specified, the standard N-EVES formulation consisted of 15 wt% HCl, 6 wt% formulated VES solution (2.4 wt% active surfactant), 0.10 wt% 15 nm SiO_2_ nanoparticles, 0.50 wt% corrosion inhibitor, and deionized water as the balance. For the VES-concentration experiments, only the formulated VES concentration was varied from 2 to 6 wt%. For the nanoparticle-concentration experiments, only the SiO_2_ concentration was varied from 0 to 0.20 wt%. For the particle-size experiments, the nanoparticle concentration was fixed at 0.10 wt%, while the nominal particle size was varied from 15 to 500 nm. All other components were maintained at the standard concentrations.

The corrosion inhibitor concentration was kept constant in the matched VES and N-EVES formulations; therefore, its independent contribution was not evaluated in this study. The hydrodynamic particle size, polydispersity index, and sedimentation stability under concentrated HCl, complete N-EVES, and spent-acid conditions were not independently determined in the present study.

### 4.2. Preparation of N-EVES System

To promote the initial dispersion of nanoparticles and reduce hard agglomeration during sample preparation, the nanoparticle-enhanced diverting acid (N-EVES) was formulated using a refined three-step method:

1. Nanofluid Preparation: A predetermined mass of SiO_2_ NPs (with average diameters of 15, 30, 100, and 500 nm) was introduced into deionized water. The mixture was subjected to 30 min of ultrasonication using an ultrasonic disperser to obtain a visually uniform nanoparticle dispersion.
2. Surfactant Integration and Self-Assembly: Under constant-temperature magnetic stirring (500 rpm, 60 °C), a specific concentration of C22^+^ VES was slowly dripped into the prepared nanofluid. Stirring was maintained for 2 h to facilitate the complete dissolution of surfactant molecules.
3. Acidification and Final Formulation: Concentrated hydrochloric acid was added dropwise under continuous stirring to adjust the final HCl concentration to 15 wt%. Concurrently, 0.50 wt% high-temperature corrosion inhibitor was incorporated. After an additional 30 min of stirring, the mixture was allowed to stand for degassing to obtain the final N-EVES diverting acid system. As a control group, a conventional VES acid system without nanoparticles was prepared following an identical procedure.

To evaluate the nanoparticle-reinforcement effect within the VES matrix, the principal rheological control was the corresponding VES acid without SiO_2_ nanoparticles. The matched VES and N-EVES formulations contained identical concentrations of HCl, formulated VES solution, corrosion inhibitor, and water, differing only in the presence or absence of SiO_2_ nanoparticles. Conventional HCl was used as the reference fluid in the rotating-disk kinetic experiments. In the core-flooding experiments, N-EVES formulations containing different nanoparticle concentrations were compared under identical operating conditions.

### 4.3. Rheological and Viscoelastic Measurements

Rheological measurements were conducted using a HAAKE MARS III rheometer (Thermo Fisher Scientific, Dreieich, Germany) equipped with a high-pressure coaxial-cylinder cell. The cell gap was approximately 1 mm, and approximately 15 mL of sample was loaded for each measurement. The sealed cell was pressurized with nitrogen to 3 MPa to prevent boiling at elevated temperatures. Steady-shear measurements were performed at 170 s^−1^ while the temperature was increased from 30 to 160 °C over 35 min, corresponding to a heating rate of approximately 3.71 °C/min. The apparent viscosity reported at 160 °C was calculated as the average over the entire 20 min isothermal holding period. Oscillatory frequency sweeps were conducted from 0.1 to 10 Hz at a fixed strain amplitude of 1%, selected within the linear viscoelastic region determined by a preliminary strain sweep from 0.1% to 100%.

### 4.4. Zeta Potential Measurements

Zeta potential measurements were performed using a Malvern Zetasizer Nano ZS (Malvern Panalytical, Malvern, UK). Direct measurement of the undiluted 15 wt% HCl formulations was not technically feasible because their high acidity and electrical conductivity exceeded the recommended operating range of the instrument. All samples were therefore diluted 100-fold with deionized water before measurement [32]. The resulting values were used only for qualitative comparison because dilution substantially decreases ionic strength, alters the electrical-double-layer thickness, and may affect micellar morphology and nanoparticle aggregation.

### 4.5. Acid-Rock Reaction Kinetics

Acid-rock reaction kinetic experiments were conducted using a high-temperature and high-pressure (HTHP) rotating disk apparatus (RDA) at 160 °C and a reaction pressure of 10 MPa. Polished marble disks were securely mounted onto the rotating shaft and reacted with conventional HCl and N-EVES systems at varying rotational speeds (1100–1500 rpm). The rotating-disk experiments used marble disks (2.54 cm diameter, 0.8 cm thickness, 5.07 cm^2^ exposed area) reacting in 500 mL of acid solution for 30 min. Following the reaction, the spent acid solutions containing H^+^ and Ca^2+^ were collected. The Ca^2+^ concentration was determined by inductively coupled plasma optical emission spectrometry (ICP-OES), whereas the H^+^ concentration was determined exclusively by acid-base titration. These measurements were used to calculate the area-normalized H^+^ consumption flux and the apparent effective H^+^ diffusion coefficient. The calculation equations and definitions are provided on the following page.

(1) $$J_{H^{+}}=\frac{(C_{H^{{+}_{,0}}}-C_{H^{{+}_{,t}}})V}{A\Delta t}$$

(2) $$D_{e,app}={\left[{1.6129J}_{H^{+}}\nu^{1/6}\omega^{-1/2}C_{H^{{+}_{,t}}}^{-1}\right]}^{3/2}$$

where $J_{H^{+}}$ is the area-normalized average H^+^ consumption flux during the reaction period (mol·cm^−2^·s^−1^); $C_{H^{+},0}$ and $C_{H^{+},t}$ are the initial and residual bulk H^+^ concentrations, respectively (mol·cm^−3^); V is the acid volume (cm^3^); A is the exposed surface area of the marble disk (cm^2^); and Δt is the reaction time (s). The calculated $J_{H^{+}}$ represents the overall acid-rock reaction rate on a proton-consumption basis rather than an intrinsic surface-reaction rate.

In Equation (2), $D_{e,app}$ is the apparent effective H^+^ diffusion coefficient (cm^2^·s^−1^), ν is the kinematic viscosity of the fluid (cm^2^·s^−1^), and ω is the angular velocity of the rotating disk (rad·s^−1^), calculated from the rotational speed N as ω=2πN/60. The coefficient 1.6129 is the reciprocal of the Levich constant 0.620 and is dimensionless. Assuming rapid H^+^ consumption at the disk surface, $C_{s,H^{+}}$ was approximated as zero, and the measured residual bulk H^+^ concentration $C_{H^{+},t}$ (mol·cm^−3^) was used as the concentration term.

### 4.6. Core Flooding and Dynamic Diversion Evaluation

To evaluate the pressure response and flow-redistribution behavior of the N-EVES formulations in carbonate cores, single-core and parallel dual-core flooding experiments were conducted using a high-temperature and high-pressure (HTHP) core flooding apparatus. The experimental temperature was maintained at 160 °C, with a confining pressure of 10 MPa and a backpressure of 5 MPa.

**Figure 14:**
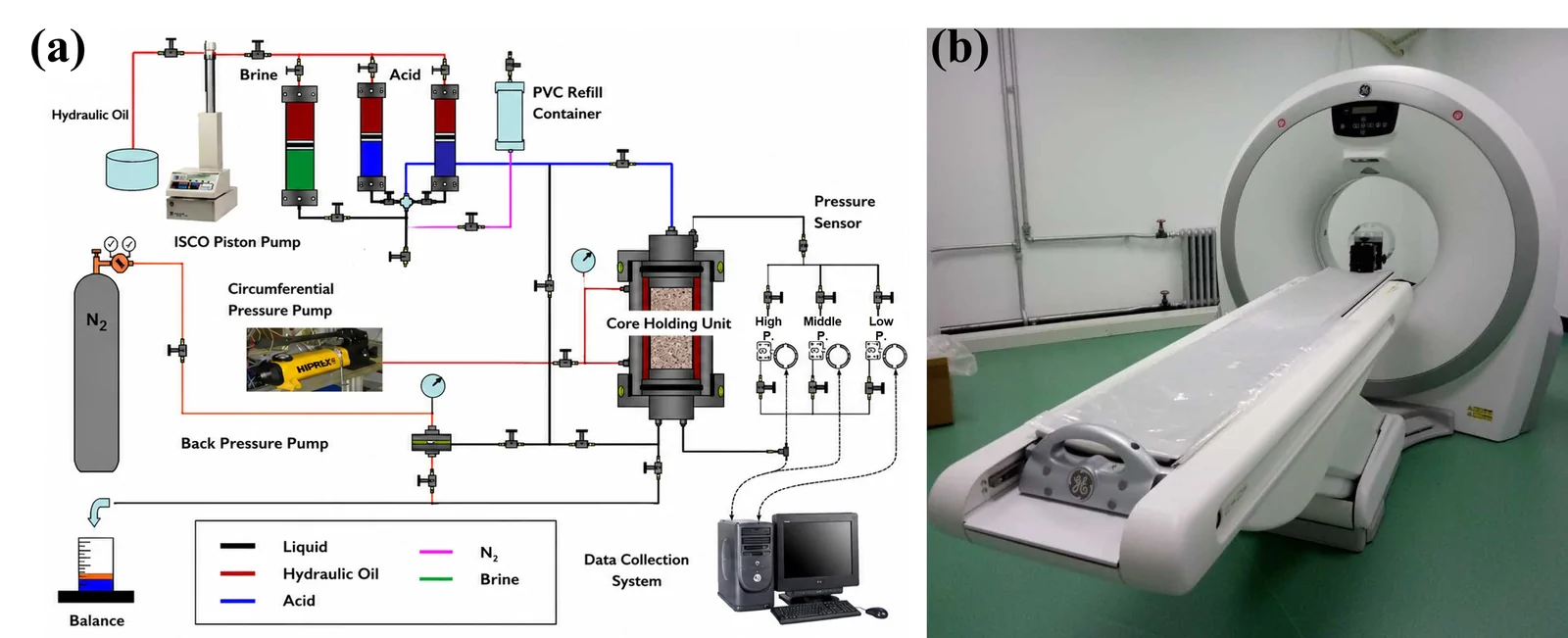
Integrated experimental setups for evaluating the intelligent diversion performance: (**a**) schematic diagram of the high-temperature and high-pressure core flooding apparatus, and (**b**) X-ray computed tomography (GE Healthcare, Waukesha, WI, USA) scanning system for 3D microscopic wormhole reconstruction.

Single-Core Flooding: Cores with varying permeabilities were injected with the N-EVES system at a constant flow rate of 1.0 mL/min. This rate was selected to provide sufficient acid-rock contact time while maintaining stable pressure acquisition and outlet-flow measurement under the laboratory conditions. The differential pressure across the core was recorded as a function of injected pore volume. The pressure-drop ratio (PDR) was defined as the differential pressure at a given time normalized by the initial differential pressure, and the maximum pressure-drop ratio (MPDR) was the maximum PDR recorded during flooding. The pore volume to breakthrough (PVbt) was defined as the cumulative injected acid volume at breakthrough normalized by the initial pore volume. The experimental schematic is illustrated in Figure 14a.

(3) $$PDR\left(t\right)=\frac{\Delta P(t)}{\Delta P_{0}}$$

(4) MPDR=max[PDR(t)]

(5) $$PVbt=\frac{V_{inj,bt}}{V_{p}}=\frac{{Qt}_{bt}}{V_{p}}$$

where Δ*P*(*t*) is the differential pressure at time t, Δ*P*_0_ is the initial differential pressure, *V_inj_*_,_*_bt_* is the cumulative injected acid volume at breakthrough, *V_p_* is the initial pore volume of the core, *Q* is the total injection rate, and t_bt_ is the breakthrough time. Breakthrough was operationally identified as the onset of a sustained sharp decrease in differential pressure following the pressure maximum. No fixed percentage threshold was imposed, and the same criterion was applied consistently to all tests.

Parallel Dual-Core Flooding: Two core holders were connected in parallel to a single injection pump. The pump supplied a total flow rate of 1.0 mL/min, which was dynamically divided between the two branches according to their instantaneous flow resistance. Each core was monitored using an independent differential-pressure transducer. The effluent from each branch was collected separately, and the outlet flow rates were determined gravimetrically using individual analytical balances. The system employed a high-permeability core of approximately 65 mD and a low-permeability core of approximately 5 mD, corresponding to a permeability contrast of approximately 13.

The outlet flow rate of each branch was calculated from the mass increase over the corresponding collection interval:

(6) $$Q_{i}\left(t\right)=\frac{\Delta m_{i}}{{\rho \Delta }_{t}}$$

(7) $$f_{L}\left(t\right)=\frac{Q_{L}(t)}{Q_{L}\left(t\right)+Q_{H}(t)}\times 100\%$$

where *Q_L_* and *Q_H_* are the outlet flow rates of the low- and high-permeability cores, respectively, Δ*m_i_* is the collected effluent mass, *ρ* is the effluent density, and Δ*_t_* is the corresponding collection interval.

The sum of the two outlet flow rates was consistent with the total pump injection rate within experimental uncertainty.

### 4.7. 3D-CT Scanning and Wormhole Reconstruction

Following the core-flooding experiments, a high-resolution micro-computed tomography (micro-CT) scanning system was employed to perform 3D scanning of the acidized cores. The reconstructed images were processed using Avizo to separate the wormhole phase from the rock matrix. In conjunction with the acid-diversion data, these 3D reconstructions were used to compare the wormhole propagation patterns and visually support the flow-redistribution results obtained from the parallel core-flooding experiments. The experimental setup is illustrated in Figure 14b. All core samples were scanned under identical conditions at 120 kV with a voxel size of 100 μm. A consistent grayscale-based segmentation procedure was applied to all reconstructed cores. The same reconstruction and threshold-selection criteria were applied to all cores to ensure comparability.

The CT reconstructions were used primarily for comparative visualization of the dominant wormhole propagation patterns. The normalized axial wormhole penetration fraction was calculated as:

(8) $$F_{ESL}=\frac{L_{W}}{L_{C}}\times 100\%$$

where *L_w_* is the maximum axial wormhole penetration length measured from the core inlet in the segmented CT volume, and *L_c_* is the total core length.

### 4.8. Microscopic Morphology and Elemental Characterization

The morphologies and elemental distributions of the nanoparticles, N-EVES samples, and treated carbonate surfaces were examined using the following characterization techniques:

1. Morphology of Nanoparticles and Dried N-EVES Samples: The pristine morphologies of SiO_2_ nanoparticles (15, 30, 100, and 500 nm) and the morphologies of dried N-EVES deposits were visualized using an FEI-F20 transmission electron microscope (TEM; FEI Company, Hillsboro, OR, USA). For sample preparation, a minimal aliquot of the suspension was deposited onto ultra-thin carbon-coated copper grids and allowed to air-dry naturally. Furthermore, high-angle annular dark-field scanning transmission electron microscopy (HAADF-STEM) combined with energy-dispersive X-ray spectroscopy (EDS mapping) was employed to perform quantitative elemental mapping (C, N, O, Si, and Cl). This approach was used to examine the spatial association and elemental co-localization of the organic VES components and inorganic SiO_2_ nanoparticles.
2. Surface Morphology and Elemental Composition after Acid-Rock Reactions: High-resolution imaging of the surface topographies of four distinct rock samples—pristine matrix, conventional HCl-etched, pure VES-treated, and N-EVES-treated—was conducted using a Quanta 200F field-emission scanning electron microscope (FESEM; FEI Company, Hillsboro, OR, USA). Prior to imaging, all rock specimens were rigorously oven-dried and subjected to gold-sputter coating. In conjunction with the FESEM, an integrated EDS spectrometer was utilized to obtain elemental spectra (Energy-keV), along with the corresponding mass fractions (wt%) and atomic fractions (at%). These data were used to compare the surface elemental compositions and assess possible adsorption or deposition after treatment with the different fluids.

### 4.9. Data Presentation

The rheological, oscillatory, and kinetic results are presented as continuous time- or frequency-dependent profiles to show the measured responses over the corresponding experimental ranges. Characteristic values, including the apparent viscosity at 160 °C and the plateau modulus, were extracted using the calculation procedures specified in the corresponding sections. Owing to the long duration, complex equipment setup, and destructive nature of the high-temperature reaction and core-flooding experiments, no independent replicate experiments were performed. Therefore, n=1 for each experimental condition, and replicate-based uncertainty and inferential statistical analyses are not reported.

## Data Availability

The data presented in this study are openly available in the article.
